# Accounting for Individual Differences in Risk of Alzheimer Disease

**DOI:** 10.1371/journal.pmed.0020082

**Published:** 2005-03-29

**Authors:** William Grant

**Affiliations:** Sunlight, Nutrition and Health Research Center (SUNARC)San Francisco, CaliforniaUnited States of America

Gatz's statement, “At least half of the explanation for individual differences in susceptibility to Alzheimer disease is genetic” [1], is, in my opinion, incorrect. As the one who led the team debating Ashford and Mortimer, whose 2002 article [2] supports this statement, at the 2001 conference on Alzheimer disease (AD) in Cincinnati (“Challenging Views of Alzheimer's Disease”) [3], I think that the evidence that dietary and lifestyle factors explain the majority of the individual risk for AD in the US is very strong. My original paper in 1997 [4] found that total dietary fat and energy intake were the most important dietary risk factors, while fish and cereal intake were the most important risk reduction factors. These findings have been generally confirmed by Drs. Luchsinger and Morris and others. The reason I did my study was that the Honolulu Heart Study reported that Japanese American men in Hawaii had 2.5 times the risk of AD of native Japanese. African-Americans have about four times the risk of AD of native Nigerians. If genetics were the primary risk factor, those living in the US would have a risk of developing AD very similar to that of individuals living in their ancestral home. The reason this is not the case is that the American diet provides too much food, which is a particular problem for those genetically predisposed to AD.
